# Retrospective Cohort Study Analysing Response to Supervised Exercise Therapy and Subsequent Revascularization in Patients with Intermittent Claudication

**DOI:** 10.3390/jcm15031037

**Published:** 2026-01-28

**Authors:** Elizabeth J. Bouch, Suzanne Austerberry, Frank L. Bowling, Steven K. Rogers

**Affiliations:** 1 Manchester Academic Vascular Research and Innovation Centre (MAVRIC), Manchester Vascular Centre, Manchester University NHS Foundation Trust, Oxford Road, Manchester M13 9WL, UK; elizabeth.bouch@mft.nhs.uk (E.J.B.); suzanne.austerberry@mft.nhs.uk (S.A.); frank.bowling@manchester.ac.uk (F.L.B.); 2 Division of Diabetes, Endocrinology and Gastrointestinal Medicine, School of Medical Sciences, Faculty of Medicine, Biology and Health, University of Manchester, Manchester M13 9PL, UK; 3 Division of Cardiovascular Sciences, School of Medical Sciences, Faculty of Medicine, Biology and Health, University of Manchester, Manchester M13 9PL, UK; 4 Division of Nursing, Midwifery and Social Work, School of Health Sciences, Faculty of Medicine, Biology and Health, University of Manchester, Manchester M13 9PL, UK

**Keywords:** intermittent claudication, supervised exercise therapy, revascularisation

## Abstract

**Background:** All major international and national guidelines recommend supervised exercise therapy (SET) for intermittent claudication (IC) as a first line of treatment, with revascularisation options to be considered for those who do not respond. Revascularisation incurs complication risks and additional costs; therefore, the need to correctly identify individuals who potentially may progress to revascularisation following SET would be of benefit. This retrospective cohort study aimed to review responses and subsequent revascularisation for individuals with IC following completion of SET. **Methods:** Retrospective data was collated for individuals who received hospital-based SET between 2016 and 2020. Demographics, Pain Onset Distance (POD), revascularisation (pre- and post-completion of SET) and quality of life (QoL) were calculated. **Results:** A total of 142 individuals were included; of those, 38 had diabetes, 48 were current smokers and 42 were female. Individuals who had a ≥75% improvement in POD were less likely to need revascularisation (*p* < 0.019). Gender, diabetes, and age did not imply likelihood of preventing revascularisation. Those who smoked were significantly less likely to go on to further revascularisation (*p* < 0.05) and those who had previous revascularisation surgery (*n* = 25) were significantly more likely to require further revascularisation (*p* = 0.0071) (32% compared with 10%). A mean positive improvement (1.77%) was seen in the EQ5D5L overall health percentage score for individuals who avoided surgery. **Conclusions:** Individuals who saw a ≥75% in POD were statistically less likely to require revascularisation post-SET. Improvements in QoL increase the probability of avoiding revascularisation.

## 1. Introduction

Peripheral arterial disease (PAD) affects 20% of people over the age of 60 in the United Kingdom (UK) [1] and presents when atherosclerosis forms flow-limiting stenosis or occlusion, resulting in insufficient blood supply to the lower limbs and/or feet. PAD is the third most common presentation of atherosclerotic disease after coronary artery disease and stroke [2]. Smoking, hypertension, diabetes, hypercholesterolaemia and ageing are recognised as risk factors [3,4]. As a result, individuals with PAD have a greater risk of heart attack, stroke and overall mortality than those without [5]. Consequently, as the population continues to age, the prevalence of PAD is expected to rise [6]. Global epidemiological projections provide important context by illustrating the scale of PAD across ageing populations worldwide. Deng et al. predict a 220% increase in PAD worldwide by 2050 [7], highlighting a widespread rise that is applicable to the UK’s ageing population.

The most common symptom of PAD is intermittent claudication (IC), described as pain on physical exertion that is relieved by rest [8]. Consequently, individuals with PAD often reduce their physical activity, leading to a sedentary lifestyle and general deconditioning [9,10], causing a faster functional decline [11]. Gardner et al. (2008) reported that individuals with PAD who engage in physical activity have a lower mortality rate than those who have a sedentary lifestyle [9]. Consequently, the promotion of exercise for these individuals is paramount.

The National Institute for Health and Care Excellence (NICE) and the American Heart Association (AHA)/American College of Cardiology (ACC) guidelines recommend supervised exercise therapy (SET) as the first-line treatment in the absence of tissue loss before revascularisation [12,13]. NICE and the UK Provision of Vascular Services document (POVS) recommends SET should involve 2 h a week of supervised exercise for a 3-month period, with individuals encouraged to exercise to the point of maximal pain [12,14]. Exercise is thought to have a positive physiological effect for individuals with PAD, including improvement in skeletal muscle metabolism, arterial collateralisation and suppression of inflammation [15]. Therefore, the aim of SET is to improve walking distances without the need for surgical intervention.

Further treatment options for individuals with IC include endovascular or open revascularisation. However, individuals with PAD frequently present with multiple co-morbidities, which increases their surgical and post-operative complication risk [16,17,18]. Although endovascular procedures are generally considered less invasive, readmission rates following elective endovascular procedures have been reported to be higher than those following open revascularisation [19]. Importantly, individuals undergoing revascularisation for chronic limb-threatening ischaemia (CLTI) or IC have a 9.8% risk of myocardial infarction (MI) or stroke and a 41.7% risk of major amputation or further peripheral revascularisation [20]. Among individuals with IC undergoing revascularisation, the 30-day risk of major amputation is 0.5%, with a mortality rate of 1% [21]. Additionally, 24-month mortality rates for individuals with PAD are approximately 6% for patients completing SET, compared with 23% following revascularisation [22]. Furthermore an overall readmission rate of 10.7% has been reported for individuals with IC following revascularisation [19]. Therefore, non-surgical management of individuals presenting with IC is vital. It is well established that PAD and the associated co-morbidities impair an individual’s walking capacity and ability to perform activities of daily living, resulting in a reduced quality of life (QoL) [16]. Consequently, treatment strategies are primarily aimed at improving these functional limitations [23]. The World Health Organisation (WHO) describes QoL as an individual’s subjective perception of their position in life, incorporating their physical health, level of independence, social relationships and psychological wellbeing [24]. Dumville et al. (2004) reported that individuals with IC experience lower QoL, particularly related to physical ability, when compared to those without claudication, reinforcing the detrimental impact of the condition on health [25]. A Cochrane review of 32 studies including 1835 participants reported that SET improved walking distances in individuals with IC, with associated improvements in QoL also reported [8]. A further study reported that individuals undergoing revascularization experienced significantly greater improvements in QoL compared with those having non-invasive treatments. However, the individuals randomised to the non-invasive treatment arm were only provided with advice to increase activity and did not attend SET, limiting the comparability of outcomes [26]. More recent evidence further supports the benefits of exercise, with a study demonstrating that 12 weeks of SET not only improved walking distance but also led to favourable changes to ankle brachial pressure indices (ABPIs), suggesting improvement in end-organ perfusion [27]. SET is generally considered a cost-effective treatment for individuals with IC [28]. Spronk et al. (2008) discussed the higher cost of endovascular revascularisation per patient, with the mean incremental cost-effectiveness ratio being 231,800 EUR/quality-adjusted life years (QALY) when compared with SET, with no statistical significance seen in the mean QALY [29]. Further research suggests that SET should be the first-line treatment due to its effective resource use through reducing early invasive revascularisation [30]. In addition to the higher costs demonstrated for endovascular revascularisation, a meta-analysis of 987 patients reported that this, as an initial treatment for individuals with stable IC, did not improve walking distances when compared with SET [31]. This combined evidence makes it difficult to justify endovascular revascularisation as an initial treatment option for this group of individuals.

Whilst it is well documented in the literature that SET improves walking distances of individuals with IC, it has not been determined whether the completion of SET reduces the need for revascularisation in the future. This study aimed to review outcomes and responses following SET for individuals with IC and the need for subsequent revascularisation. It is hoped that this will improve clinical decision-making and empower individuals upon completion of SET.

## 2. Methods

Retrospective databases and electronic health records were accessed from a single major tertiary arterial centre for individuals with IC who completed SET. All individuals who completed SET from 2016 to 2020 were initially included; however, if there was an incomplete data set in terms of outcome measures, they were later excluded, as appropriate analysis could not be undertaken. Individuals who failed to attend the full 12 weeks of SET were excluded during data cleaning. Using the Health Research Authority decision tool, this study was not deemed to be research; therefore, it was classed as a service evaluation and further approval was not required. Furthermore, as this study was a retrospective data collection using non-identifiable data, consent was not required.

### 2.1. SET Standard of Care

Individuals were referred to SET following a diagnosis of PAD by an ankle brachial pressure index (ABPI) or an arterial duplex scan, which were performed within the secondary care setting. Whether an ABPI or arterial duplex was performed depended on symptomology and severity, based on the referrer’s clinical judgement. Individuals described symptoms of IC in one or both legs and none had active ulcers or were in rest pain.

A functionally graded-treadmill test and a QoL questionnaire were collected at initial and final SET assessment. The graded treadmill test is a standardised assessment tool and is used to determine the primary outcome measures of Pain Onset Distance (POD) and Absolute Claudication Distance (ACD). The Gardner–Skinner protocol was utilised, which sets the speed at 3.2 km/h with the gradient increasing by 2% every 2 min [32]. The individual was asked to inform the clinician of when their pain started (POD) and when they reached maximum pain and needed to stop (ACD). The EQ5D5L was used to assess QoL as per NICE recommendations and used as a secondary outcome measure [33].

Individuals attended SET, which was run by a Physiotherapist and a Vascular Specialist Nurse, once a week for 1 h over 12 weeks. Whilst this does not achieve the NICE recommendation of 2 h a week, patients were counselled to supplement the class with a home exercise programme. This was not objectively measured and is therefore recognised as a limitation of the study, but reflects the real-world analysis that was completed. The class itself consists of 8 lower-limb-based exercises: tip-toe walking, step-ups, walking, heel raises, marching, cycling, treadmill walking and sit-to-stand. Each exercise was completed for up to 3 min, aiming to work to maximal pain, with individuals being monitored throughout the programme. When individuals managed to complete the 3 min of exercise with less pain, the exercise complexity was increased, ankle weights were introduced, and the walking speed/gradient was increased on the treadmill.

### 2.2. Outcome Measures

Age (at completion of SET), gender, smoking and diabetes status were recorded (Microsoft Excel, Microsoft Inc., Redmond, WA, USA, Version 2406). Smoking status was divided into current or non-smoker, with ex-smokers included in the latter. In addition, POD, ACD, QoL via EQ5D5L and whether individuals had revascularisation pre- or post-completion of SET were recorded. Data was collected from electronic health records, including clinic and discharge letters, operation notes and retrospective databases. Revascularisations/further surgery included angioplasty (with/without stenting), endarterectomy, bypass, and minor and major amputation. Following initial analysis, patients were then separated into 4 groups:

- Group 1: No surgical intervention (*n* = 105)
- Group 2: Surgical intervention prior to SET (*n* = 17)
- Group 3: Surgical intervention post-SET (*n* = 12)
- Group 4: Surgical intervention pre- and post-SET (*n* = 8)

### 2.3. Statistical Analysis

A statistical package (R version 4.2.1, 2022, The R Foundation for Statistical Computing) was used for analysis. Power calculations were not completed due to the retrospective nature of the study. It is recognised that the small sample sizes in some of the groups mean some calculations may be underpowered. Binomial generalised linear models (GLMs) were used to determine if the measured changes in distances walked and EQ5D5L were significantly linked to a successful outcome for the individual. Success of the class was defined as not requiring revascularisation following completion of SET, with a significance threshold of *p* = 0.05.

## 3. Results

One hundred and forty-two individuals completed SET (female *n* = 42), with a mean age of 66.8 years (s.d. = 9.9), with >70% of patients being between 56 and 75-years old. An ex-smoker was classed as a non-smoker, with a third of individuals (*n* = 48) being current smokers, and approximately a quarter had diabetes (*n* = 38). Individuals who were current smokers were statistically less likely to require revascularisation/further surgery (*p* = 0.0274), with only two individuals who smoked going on to have revascularisation on completion of SET. Gender, age and diabetic status had no statistical effect on the likelihood of future revascularisation.

### 3.1. Failure to Complete SET

Eighty-five individuals failed to complete SET. The two main reasons for not completing the class were not attending (DNA), which accounted for 55% (*n* = 47), and being unwell with other medical or musculoskeletal problems at 21% (*n* = 18). Additionally, six individuals requested to have a follow-up appointment with a Consultant to discuss other options, one died, eight declined SET and five continued with exercise in the community. As the study aimed to review outcomes and responses to completing SET, this group of individuals was not included in further data analysis.

### 3.2. Walking Distance

#### 3.2.1. Pain Onset Distance

Table 1 shows the median improvement in POD, with groups 1 and 2 showing the greatest improvement. Individuals who saw an ≥50% (*p* = 0.06), ≥75% (*p* = 0.019) or ≥100% (*p* = 0.022) improvement in POD were statistically less likely to require revascularisation, with significance being reached for the greater improvements. Non-smokers had a slightly worse improvement in POD 171% (115 m) following SET compared to current smokers 230% (126 m).

#### 3.2.2. Absolute Claudication Distance

Table 2 reports the median improvements in ACD; similarly to POD, groups 1 and 2 show the greatest improvements. All four groups have an overall percentage improvement in ACD, with groups 3 and 4’s improvement being less than 50%.

**Figure 1:**
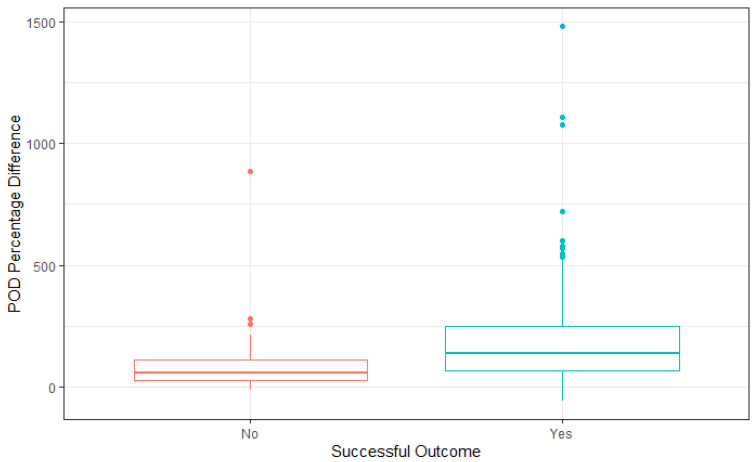
Changes in POD compared with SET outcome.

Differences in POD from initial to final assessment were compared with successful outcomes (Figure 1). Patients who had a successful outcome following SET had a greater percentage (136%) improvement in POD than those who had revascularisation (59%).

### 3.3. Previous Revascularisation

Twenty-five (18%) individuals had previous revascularisation prior to starting SET. They were statistically more likely to require further revascularisation/further surgery (*p* = 0.0071), with 32% going on to have revascularisation, compared to 10% in the group who had no prior revascularisation. Three individuals went on to have a major amputation; all were in group 4, having had intervention prior to attending SET. The time to amputation following completion of SET was 5 years for two individuals and 4 years for one.

### 3.4. Quality of Life

**Figure 2:**
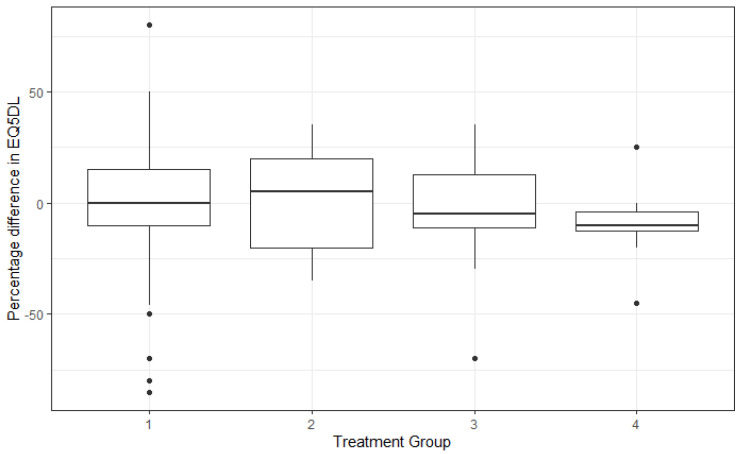
Change in overall quality of life per group. Group 1: No surgical intervention. Group 2: Surgical intervention prior to SET. Group 3: Surgical intervention post-SET. Group 4: Surgical intervention pre- and post-SET.

The mean positive QoL improvement in groups 1 and 2 was 1.77% (s.d 25.1) for the EQ5D5L overall health percentage score, compared with those in groups 3 and 4 having a 6% mean negative change (s.d. 24.9) (Figure 2).

**Figure 3:**
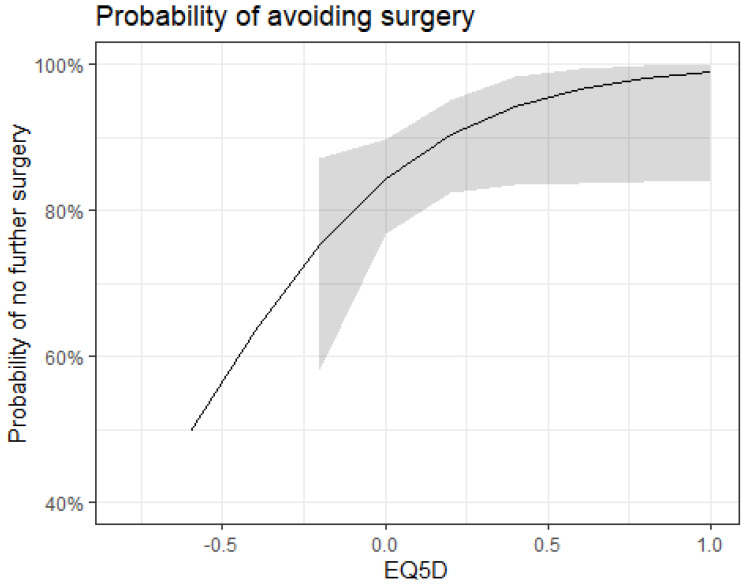
Logistic regression of avoiding surgery compared to changes in EQ5DL.

Those who saw a positive improvement in the EQ5D5L functional domain were more likely to have a successful outcome, although this was not significant (*p* = 0.54). Those individuals who had revascularisation saw minimal change in their EQ5D5L functional domain. As improvement in the EQ5D5L score increased, the logistic regression showed a reduction in the likelihood of requiring further surgery (Figure 3).

## 4. Discussion

In this study we identified improvements in walking distances among individuals with IC following completion of SET, as well as changes in QoL and demographic factors that may inform the need for revascularisation. Those with an improvement in their POD ≥75%, following completion of SET, were statistically less likely to require revascularisation. Improvements in QoL also increased the probability of avoiding revascularisation.

During the data collection period, 227 individuals started the 12-week SET programme. It is acknowledged that adherence to SET for individuals with IC is poor. In this study 37% of individuals who started SET failed to complete the full programme, which is higher than the 25% previously reported [34]. Our patients come from across our region, covering a large geographical area, which may account for the slightly higher DNA rate. A recent national audit reported that only 6.8% of Vascular Centres in the UK offered SET that was compliant with NICE guidelines [35]. The lack of resources is likely to affect adherence to attending and completing SET; therefore, it would be beneficial to address the provision of SET nationally, so it is readily accessible in the local area for individuals who require it. Those individuals who failed to complete SET were excluded from the study; it is recognised that this potentially introduces selection bias, as this group of individuals may be less motivated and have a poorer level of function. However, this study aimed to review the outcomes for those who did complete SET; therefore, it was deemed out of the scope of this retrospective evaluation to analyse non-attenders. Those who did complete the programme all attended for twelve 1 hour sessions; we understand this does not meet NICE guidelines, but this reflects the national trend of services being non-compliant, and benefits were still observed. Individuals were advised to complete the exercises a further 2–3 times a week at home; however, we were unable to standardise or objectively measure their activity, which introduces a confounding variable that could have affected the magnitude of the results. It may be useful to consider this in the future to ensure consistency.

Additionally, we identified that individuals who smoked were less likely to require revascularisation. Smoking is associated with a 48% increase in 30-day post-procedural complications following revascularisation compared to non-smokers [36]. This underlines the NICE PAD clinical guideline recommendation that revascularisation should only be offered to individuals after reinforcement of the benefits of modifying risk factors and an unsatisfactory outcome following SET [12]. However, caution must be taken when interpreting this result, as the need for revascularisation is likely to have been affected by clinical decision-making bias, rather than smoking reducing the requirement. Gardner et al. (2004) reported similar improvements in walking distances for smokers and non-smokers following SET [37]. Due to the higher variability in outcomes for smokers, we reported a slightly greater improvement in POD for current smokers, 230% (126 m), compared to none smokers 171% (115 m), following SET, although this was not significant. Whilst smoking can be detrimental to surgical outcomes, it does not appear to impact the benefits individuals see following SET. This is likely due to exercise being widely accepted to improve overall fitness and reduce the risk of major adverse cardiovascular events. The class setting can have the added benefit of regular smoking cessation counselling and peer support for those individuals who are trying to stop.

However, there are challenges to assessing exercise; therefore, a universal graded-treadmill test protocol for the assessment of walking distance was required. This study used the Gardner–Skinner protocol, with some limitations identified [38,39]. As this study was based in clinical practice, a maximum time of 10 min was adopted for the treadmill test due to time restrictions during a class setting. This is not ideal, but necessary when numerous individuals need to use the treadmill throughout the duration of the class. Therefore, those individuals who were able to walk for the maximum time at both initial and final assessment would show no improvements in ACD. This represents a ceiling effect with ACD; however, for these individuals, the severity of pain and POD had often improved. POD, therefore, had greater utility and was a predictive outcome for improvements in walking distance.

As an adjunct to the above, it was necessary to calculate walking distance in terms of both distance (metres) and as a percentage improvement. A systematic review on the benefits of exercise utilised percentage change in walking distances rather than distance or time, as it provides a more holistic approach to analysis [8]. An improvement of 50 m for an individual whose baseline was poor initially is much more likely to improve QoL and function compared with an individual who is able to walk much further on initial assessment. To compare individuals and identify factors associated with revascularisation, it was therefore necessary to calculate the percentage change in this study. We reported that those individuals who had a successful outcome following SET had greater improvements (percentage and distance) in POD, with groups 1 and 2 reporting the biggest improvements. The percentage change for both groups 1 and 2 was >75%, which was reported as significant for not progressing to revascularisation at a later point. Groups 3 and 4, who both went on to have revascularisation, have the smallest increases (46% and 76%, respectively) in POD.

It is worth noting that overall, all four groups showed some improvements in their POD. Prehabilitation is a strategy aimed at beginning rehabilitation prior to surgery and managing risk factors in order to improve postoperative outcomes [40]. Currently, evidence surrounding the effectiveness of prehabilitation for individuals undergoing vascular surgery is limited, with a systematic review reporting promising results for individuals undergoing elective aortic aneurysm repairs; unfortunately, no studies were found for those undergoing lower-limb revascularisation [41]. However, prehabilitation has been shown to improve outcomes following abdominal and orthopaedic surgery [42,43]. Therefore, this could imply that SET should be considered as a first line of treatment for individuals with IC, as it could be considered as part of a prehabilitation programme. Those individuals in groups 3 and 4 who went on to have revascularisation had improved functional ability after completion of SET. It was not in the scope of this study to review postoperative outcomes for groups 3 and 4, but this may be of interest in the future.

Previous evidence has reported that individuals who complete SET as an initial treatment for IC undergo fewer revascularisations than those who have had an initial invasive treatment [44,45]. This is similar to the results of this study, which support this evidence. Although this is important, identifying which individuals may require revascularisation due to failed SET is beneficial, as we know that earlier revascularisation is often accompanied by technically simpler interventions. We identified that 10% of individuals go on to have revascularisation if completing SET as the first line of treatment, compared to 32% who had previous revascularisation before completing SET. The complexities, timescales, and reasons for revascularisation for groups 3 and 4 were not explored in this study, and it is acknowledged that this is a clinical decision based on individual needs and surgeon preferences. It was reported that three individuals had major amputations following completion of SET; therefore, it can be assumed that they went on to develop chronic limb-threatening ischaemia rather than solely symptoms of IC. All three of these individuals had a previous intervention prior to starting SET; therefore, it is likely they had a complex vascular history with multiple attempts to improve vascular supply. Interestingly, the time from completing SET to amputation was 5 years for two individuals and 4 years for one, which may reflect a clinically meaningful delay in progression to amputation. In the future, percentage improvement in POD could be used to clinically assess the need for revascularisation.

Interestingly, a recent meta-analysis compared outcomes of individuals with stable IC whose first line of treatment was SET, endovascular revascularisation, or a combination of the two treatments. It was reported that the latter showed significant improvement in walking distances when compared with SET alone [31]. This suggests the possible need to consider endovascular revascularisation as an adjunct to SET. In this study, group 2 had undergone revascularisation prior to SET; however, it was not analysed whether this was open or endovascular, and the time to SET following revascularisation was not immediate. This group did report good improvements in walking distance, but due to the above limitations, direct comparisons cannot be made in this instance.

Furthermore, the need for revascularisation is often due to pain, with previous evidence reporting that IC pain has a significant negative impact on QoL, with the ultimate goal for treatment being to improve this [46]. Therefore, measuring changes in QoL when determining the effect of SET for IC is clinically important. The current literature predominantly uses the Short Form 36 (SF-36) as a generic QoL outcome measure, reporting statistically significant improvements in QoL after 12 months [47]. The EQ5D5L was utilised in this study as it is quick and easy to administer in clinical practice and recommended by NICE [12]. We demonstrated a positive change in the EQ5D5L of 1.77% for individuals who did not go on to have revascularisation and a negative change of 6% for those who went on to have revascularisation. This reinforces the perception that an individual’s QoL is affected by their functional ability and ultimately the need for revascularisation.

As this study was a retrospective data analysis, we were unable to manage cofounding variables such as clinical decision making and adherence to SET and home exercise. Assessments and treatment were carried out in a clinical setting; therefore, some limitations are acknowledged. These include the ceiling effect being reached for ACD on the graded-treadmill test and the fact that the SET offered does not reach the NICE recommendation of 2 h/week. This real-world analysis also meant that endpoint duration differed amongst individuals (mean 5.3 years), with a minimal follow-up time of 4 years. Our results were still significant, which may not have been identified within the confines of an RCT and fixed-timepoint calculations. However, it is worth noting that due to this, the number of individuals undergoing revascularisation may be under-reported, but this would similarly affect all four groups. Furthermore, the scope of this study was to evaluate outcomes for those individuals completing SET. It may be beneficial in the future to review surgical outcomes for those who fail to adhere to SET and the reasons for non-adherence.

Only 14% of individuals completing SET required further revascularisation, which supports the NICE recommendations that it should be considered as a first line of treatment for individuals with IC. The results from this study have been used to alter local clinical practice and could provide further insight to aid clinicians’ decision-making. The study suggests a benchmark as to what a good outcome may be for an individual in terms of percentage increase in POD, as revascularisation should only be offered if a satisfactory outcome is not achieved through SET [12].

## 5. Conclusions

Outcome measures of walking distances and QoL questionnaires can be useful tools to inform decisions regarding revascularisation post-SET. Changes in POD and EQ5D5L may be useful in assessing the likelihood of further revascularisation, with individuals demonstrating ≥75% improvement in POD and improvements in EQ5D5L appearing less likely to require revascularisation. This can ultimately aid clinical decision-making for individuals with IC. It may be beneficial for future research to investigate the outcomes of individuals who fail to attend SET and whether the site and/or severity of disease impacts the functional outcomes of SET. Resources for SET remain limited within the UK; therefore, this would allow clinicians to offer SET to those individuals who are likely to gain the most benefit.

## Figures and Tables

**Table 1:** Changes in POD from initial to final assessment.

Group	Median (m)	Median (%)	IQR
1: No surgical intervention	109	143	181
2: Surgical intervention prior to SET	75	108	90.5
3: Surgical intervention post-SET	51	46	102
4: Surgical intervention pre- and post-SET	48	76	102

**Table 2:** Changes in ACD from initial to final assessment.

Group	Median (m)	Median (%)	IQR
1: No surgical intervention	121	69.4	159
2: Surgical intervention prior to SET	91	126.3	156
3: Surgical intervention post-SET	10.5	4.25	120
4: Surgical intervention pre- and post-SET	92.5	49.33	68.25

## Data Availability

The original contributions presented in this study are included in the article/ Further inquiries can be directed to the corresponding author.
